# Supplementary material for: The nematode Caenorhabditis elegans and the terrestrial isopod Porcellio scaber likely interact opportunistically
Source: PLoS One. 2020 Jun 26;15(6):e0235000. doi: 10.1371/journal.pone.0235000 (PMC7319334; doi:10.1371/journal.pone.0235000)
Supplement: S1 Fig — Isoamyl alcohol (diluted to 1:1000 in ethanol) and 1-octanol serve as control attractant and control repellant, respectively, for both the N2 (left) and PB306 (right) C. elegans strains. Both strains respond neutrally to the three solvents used for the isopod washes (S1 and S2 Tables). Error bars are standard deviation. (DOCX) [file pone.0235000.s001.docx]

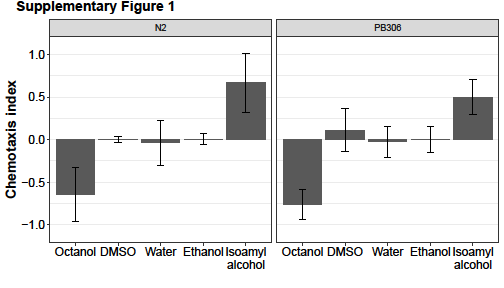


**Supplemental Figure 1**. Isoamyl alcohol (diluted to 1:1000 in ethanol) and 1-octanol serve as control attractant and control repellant, respectively, for both the N2 (left) and PB306 (right) *C. elegans* strains. Both strains respond neutrally to the three solvents used for the isopod washes (Supplemental Table 1, Supplemental Table 2).
