# Supplementary material for: The nematode Caenorhabditis elegans and the terrestrial isopod Porcellio scaber likely interact opportunistically
Source: PLoS One. 2020 Jun 26;15(6):e0235000. doi: 10.1371/journal.pone.0235000 (PMC7319334; doi:10.1371/journal.pone.0235000)
Supplement: S3 Fig — Yellow bars represent extractions prepared from male isopods. Red bars represent extractions prepared from female isopods. Chemotactic indices from all isopod extractions were not significantly different from the chemotactic indices of its corresponding neutral control (S1 Table). Significance scores (p values) are in S5 Table. Error bars are standard deviation. (DOCX) [file pone.0235000.s003.docx]

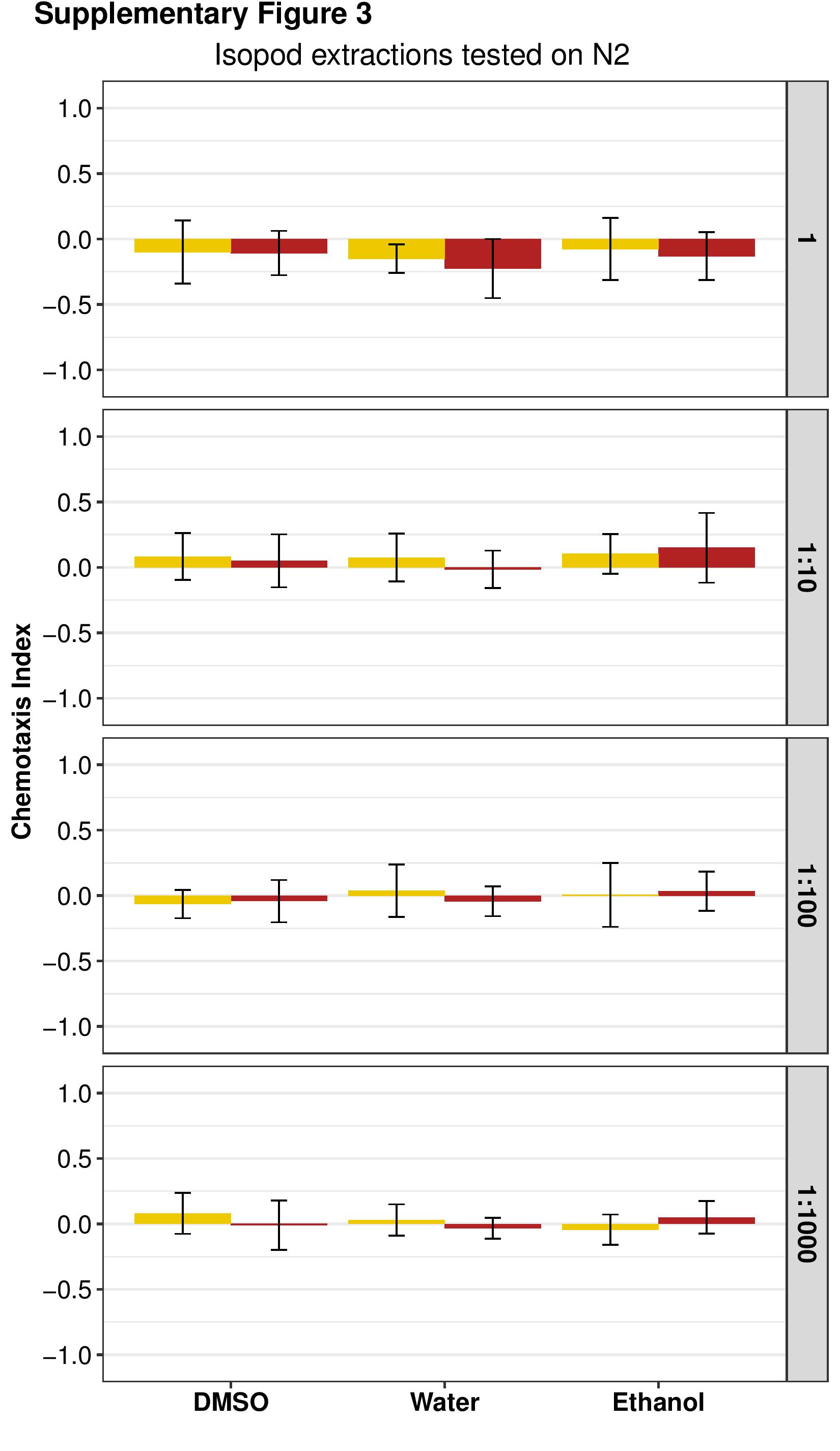


**Supplemental Figure 3.** C. elegans N2 adults respond neutrally to P. scaber extractions at four different dilutions of the initial extraction (1, 1:10, 1:100, and 1:1000). Yellow bars represent extractions prepared from male isopods. Red bars represent extractions prepared from female isopods. Chemotactic indices from all isopod extractions were not significantly different from the chemotactic indices of its corresponding neutral control (Supplemental Table 1). Significance scores (p values) are in Supplemental Table 5.
