# Supplementary material for: The nematode Caenorhabditis elegans and the terrestrial isopod Porcellio scaber likely interact opportunistically
Source: PLoS One. 2020 Jun 26;15(6):e0235000. doi: 10.1371/journal.pone.0235000 (PMC7319334; doi:10.1371/journal.pone.0235000)
Supplement: S4 Fig — Yellow bars represent washes prepared from male isopods. Red bars represent washes prepared from female isopods. Chemotactic indices from all isopod washes were not significantly different from the chemotactic indices of its corresponding neutral control (S3 Table). Significance scores (p values) are in S5 Table. Error bars are standard deviation. (DOCX) [file pone.0235000.s004.docx]

**
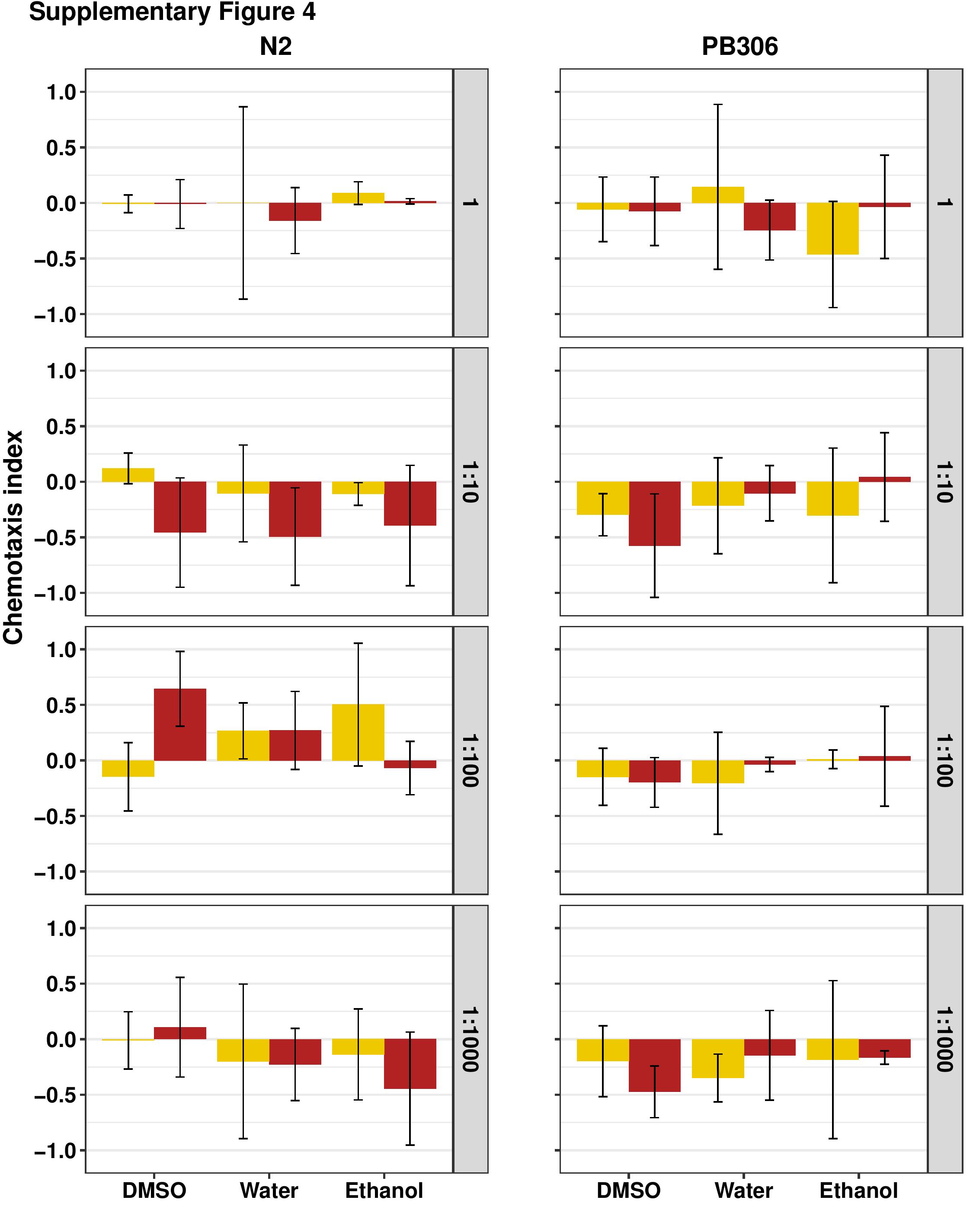
**

**Supplemental Figure 4.** C. elegans N2 and PB306 dauers respond neutrally to P. scaber washes at four different concentrations (1, 1:10, 1:100, and 1:1000). Yellow bars represent washes prepared from male isopods. Red bars represent washes prepared from female isopods. Chemotactic indices from all isopod washes were not significantly different from the chemotactic indices of its corresponding neutral control (Supplemental Table 3). Significance scores (p values) are in Supplemental Table 5.
