# Supplementary material for: The nematode Caenorhabditis elegans and the terrestrial isopod Porcellio scaber likely interact opportunistically
Source: PLoS One. 2020 Jun 26;15(6):e0235000. doi: 10.1371/journal.pone.0235000 (PMC7319334; doi:10.1371/journal.pone.0235000)
Supplement: Attachment — Submitted filename: Responses_Archeretal.pdf [file pone.0235000.s011.pdf]

We thank the reviewers for their comments and critiques. Our comments are in italics below. We believe the manuscript is much improved.

Reviewer #1: The manuscript by Archer et al. explores the relationship between the nematode Caenorhabditis elegans and the isopod Porcellio scaber. This study investigates if the nematode is (specifically) attracted to odors and compounds present on the isopod, possibly important for phoresy. The nematode-phoront relationship is studied for two wild isolates of C. elegans: the labstrain N2 and the wild-strain PB306 – that has been isolated from a P. scaber isopod. Choice assays between isopod extractions and diverse control solvents are used to investigate if C. elegans is attracted to the isopods odors. This study provides an interesting perspective on the relation between C. elegans and its phoronts and gives insights in natural processes that could be important in the (local) spread of C. elegans nematodes.

Major points:

• This study shows a negative, which we think is not a problem and actually enriching to the literature. But, it is hard to prove a negative. Hence, we would suggest altering the title and the tone at some places in the manuscript. Actually, we think the running title is better. Given that C. elegans nematodes in the wild have been repeatedly found associated with isopods both in literature and as listed on Cendr; we think it is acceptable to trust that an association can exist. The problem with proving a negative should become explicitly clear in the discussion (again, no reason not to publish this paper; but it's important to stress the particular conditions that this study was executed in). In the detailed comments some suggestions are listed.

We greatly appreciate this comment. The title has been updated to "The nematode Caenorhabditis elegans and the terrestrial isopod Porcellio scaber likely interact opportunistically" to match the old running title and make the negative result less definitive. Additionally, throughout the manuscript (as noted below), we tried to soften the definitive language.

• The paper suggests that C. elegans nematodes are not specifically attracted to P. scaber isopods, however we think this cannot be concluded from the use of chemical isolates only. Other factors besides odors and chemicals (possibly also unknown ones) could play a role in host seeking behavior. Therefore, limitations of the method used to investigate attraction should be critically discussed and this discussion should be reflected throughout the paper text.

We have added the following paragraph to the discussion (Lines 221-231). We believe that this paragraph along with some more description of the isopod source should address this concern.

"The isopods used in this study were collected from the wild and then grown in the laboratory in a controlled and sterile environment. It is possible that the change in environment could alter signals affecting chemoattraction in the wild. Our general assumption, that PB306 would likely show an attraction to isopods, was clearly not supported in this study. However, if the relevant cues come from the natural environment then it is not surprising that, when a wild strain is placed into the unnatural and sterile laboratory setting, the behavior would change as well. Additionally, given that PB306 did not display the expected behavior, we chose not to test it for chemoattraction to whole isopod extracts. Moreover, other factors besides odors and chemicals could play a role. For example, some nematode ascarosides promote aggregation among individuals and sex pheromones act as attractants (Simon & Sternberg, 2002; White et al., 2007; Srinivasan et al., 2012)."

• Although the authors clearly introduce that N2 is a lab-adapted strain that may have lost specific host-seeking behavior, they test the preference for whole-isopod extracts only in this strain. As such conclusions cannot be generalized to other wild-type genetic backgrounds (such as PB306).

We have addressed this in the Discussion by adding the text (also part of the paragraph quoted above) "Our general assumption, that strain PB306 would likely show an attraction to isopods, was clearly not supported in this study. However, if the relevant cues come from the natural environment then it is not surprising that when a wild strain is placed into the unnatural and sterile laboratory setting the behavior would change as well. Additinonally, given that PB306 did not display the expected behavior, we chose not to test it for chemoattraction to whole isopod extracts."

• None of the figures shows or mentions replicates (biological or technical). Although it is deducible from the supplemental tables that the experiments were replicated properly, the numbers tested should be mentioned in the text as well.

We have added the values for n into the figure captions.

• The choice for presenting the 1/1,000 dilution as the main result is not clearly substantiated, going back-and-forth between the supplementary figures and the main figures, this was confusing.

We added the following statement to the Methods (Lines 294-294): "Because all tested dilutions did not affect chemotaxis, figures in the main text depict results for the 1:1000 dilution only as this concentration had the smallest variance."

Detailed comments:
• It would have been easier if authors had included page numbers and line numbers for making comments.

We have made this change.

Introduction
• The second sentence (first paragraph) is strangely formulated and confusing.

Thank you for this edit. We changed the text to: "The relationship between phoront and host is commensal. Phoronts can be generalists using numerous host species or specialists with a single or few specific hosts."

• The third sentence (first paragraph): should it not be '... distance by their...'?

We changed the text to: "Most phoronts are animals that have a limited ability to travel any significant distance by their own power."

• The sentence describing the paper by Petersen et al 2015 seems to suggest that they studied chemo attractants of slugs and snails, because of the connection to the previous sentence, but this is not the case.

We understand the confusion and have updated the text (Lines 76-82): "An association with slugs and snails has also been observed with C. elegans, where nematodes have been recovered from the intestines and feces of Arion sp. slugs (Chen et al., 2006; Petersen et al., 2015). Moreover, previous studies have shown that

mucus of the slug Arion subfuscus and the snail Helix aspersa act as strong chemoattractants for P. hermaphrodita (Rae et al., 2009; Andrus & Rae, 2019). Taken together, these observations suggest that C. elegans might also detect and move toward chemical cues from invertebrates that act as hosts for dispersal."

• Last sentence second paragraph: ', other life stages have been isolated as well..'

We have changed the text to read "Additionally, although dauer larvae are the life stage most frequently found in association with invertebrates, other life stages have been isolated as well."

Results

• Page 11 'as such it is likely to have invertebrate': change 'it' to 'PB306' to prevent confusion.

We have made this change.

• Page 11 '...to hypothesize that these strains...': change 'these strains' to 'N2 and PB306'

We have made this change.

• Page 11 'nonpolar compounds were used'

The reasoning behind the choice of solvents was described further in the Results and Methods

• Page 15 describes that PB306 was weakly repulsed, but no test for significance or p-value is mentioned.

The p-value was added into text.

Figures

• A general method figure would help understanding the experiments performed more easily.

In the methods section, we have added more explicit information for a video and the accompanying paper (already cited in the text) depicting the general method "See https://www.jove.com/video/50069/c-elegans-chemotaxis-assay for a step-by-step visual guide and overview of the experimental methodology."

• Fig 2/Fig S4 The variation in dauer larvae choice assays appears to be larger than the variation measured for adults. Could the authors discuss or explain the difference? Moreover, it would be good to show the variation in control experiments like for Fig S1.

We added the following statement to the text (Lines 172-176): "Moreover, the variance among dauer individuals appears to be higher than for adults in the corresponding assay. This result was not directly tested because we found no clear attraction to P. scaber. The increase in variance could be caused stage-specific differences in behaviors between dauers and adults in the absence of food, as was found previously (Casada & Russell, 1975; Avery & Horvitz, 1990; Gaglia & Kenyon, 2009)."

Discussion

• The first paragraph of discussion does not seem to link previous findings to this study. Instead it discusses previous findings (by Lee et al 2012 and 2017) without clear connection to the current findings.

We changed wording to connect the previous results to our current findings as follows (Lines 211-220):

"Using chemotaxis assays, our results demonstrate that C. elegans is neither attracted to nor repulsed by chemical cues from the isopod P. scaber in laboratory-based chemotaxis assays. These results suggest that C. elegans phoresy might not be directed toward the terrestrial isopod P. scaber but is instead opportunistic. Consistent with this hypothesis, Lee et al. (2012) showed that the dauer-specific behavior, nictation, is both an opportunistic behavior towards a phoretic host and is necessary for dauer individuals to disperse via the fruit fly Drosophila melanogaster (see Lee et al. (2017) for a discussion of the genetic basis underlying variation in this behavior in natural populations). However, this result does not preclude the possibility of chemoattraction to preferred hosts at other life stages but did demonstrate that for dauer individuals dispersal only happened when nictation behavior was present."

• Where does the observation that dauers are most commonly associated with invertebrates without a chemical cue come from? No references were added to that statement. Also, if more is known about chemical cues of vectors in general it would be interesting to discuss these. Or mention that little is known.

We have removed that sentence because we could not find a relevant reference for C. elegans.

• What type of chemicals could be attractants for nematodes? Is anything known about their chemical composition?

We have added the statement to the discussion (Lines 228-231): "Moreover, other factors besides odors and chemicals could play a role. For example, some nematode ascarosides promote aggregation among individuals and sex pheromones act as attractants (Simon & Sternberg, 2002; White et al., 2007; Srinivasan et al., 2012)."

• The origin of the nematodes is clearly discussed, but not that of the isopods. Could they be adapted to the lab themselves? Additionally, perhaps these isopods are smelled in natural situations by the nematodes because of the organisms they associate with or produce different cues because of the (fresh) food they eat. Could it be that lab-grown sterile animals lack scent? The potential effects of using isopods in a laboratory setting should be discussed.

Thank you for this suggestions. We added to Methods "Upon each order, isopods were collected from nature just prior to being shipped and therefore do not harbor any lab adaptation." and to the Discussion "It is also possible that the change in isopod diet from a wild food source to the sterilized leaves provided in the lab altered signals affecting chemoattraction in a natural setting."

• The study uses dauers 48h after induction; however from other nematode species (e.g. plant-parasitic cyst nematodes) it is known that older dauers react differently to environmental cues. It is possible that the same is true for C. elegans (although I'm not aware of literature investigating this).

We have added the statement "It is possible, however, that dauer animals respond differently in a manner dependent on the amount of time spend in the dauer stage but we are not aware of any literature addressing this point in C. elegans."

Material and methods
• 'PB306 and N2.' This is not a sentence.

We have made this edit.

• P. scaber natural origin is not described. Are these wild or lab animals? Where do they occur? Global or local?

We have added the following "Upon each order, isopods were collected from nature just prior to being shipped and therefore do not harbor any lab adaptation."

• Unclear if there is any age synchronization or selection criterium for the isopods selected for the experiments.

We have added the statement "No additional selection beyond verification of sex characteristics was done."

Reviewer #2: The authors describe some experiments to test if C. elegans is chemically attracted to the isopod P. scaber, given elegans has been collected from P. scaber in the past, and can use P. scaber for dispersal.

Two nematode genotypes are tested, the lab adapted N2 reference and a wild isolate collected from isopods, PB306, as adults and as dauer larvae. Isopods are tested by sex. Standard plate-based attraction assays are used, testing volatiles from live isopods, and polar and non-polar extracts.

The experiments are well described and sufficiently replicated, and the results are decisively against any strong, unconditional attraction, which is the primary hypothesis of interest.

I'm not convinced the statistical analysis makes full use of the data, but it is clear that the main conclusion will not be sensitive to method. Whether there are significant effects of genotype (PB306 appears to be generally more repulsed than N2, perhaps), or of the many other tested factors, would be better answered by a joint (e.g., binomial linear model using the raw counts), rather than pairwise, analysis.

We agree with the reviewer. We noticed the possibility that PB306 might be more repulsed by isopod washes/extraction as well, However, we wanted to specifically test attraction, and that result was clearly not supported. We decided to forgo additional analyses that were beyond the original tested hypotheses of this study. As stated, the main conclusion about the lack of attraction is not sensitive to method.

I have made some minor comments on interpretation and communication in the commented pdf.

We attempted to respond to the comments in the PDF below.

1. *Chen et al reference added*

2. Reviewer - “This seems overly reticent. I think it's safe to say that any consistent association between N2 and any other form of life beyond it's food has been well and truly abolished by lab culture.”

   We have edited the text (Lines 99-103) “Because N2 has been removed from essentially all natural ecological interactions during its domestication in the lab and raised almost exclusively in association with Escherichia coli as a food source, it is likely genetically and behaviorally distinct and does not resemble most of its wild counterparts (Persson et al., 2009; McGrath et al., 2011; Duveau & Félix, 2012; Andersen et al, 2014; Sterken et al., 2015).”

3. Reviewer - “any "potential" invert-associated traits are likely to be preserved”

   We edited the text to add “potential”. “As such, it is likely to have potential invertebrate-associated traits intact…”

4. Figure 1
   a. *Changed caption to specify adults*
   b. *Changed “respond neutrally” to “do not respond”*
   c. *Added number of trials (n = xx) and info about error bars*
   d. *Changed caption notation to be less repetitive (changed Figure 2 in a corresponding manner)*

5. Reviewer - “this is unfortunate, given the expectation that PB306 is more likely then N2 to respond.”

   Relevant text - “Because no significant effects of extractions were observed, we did not test the PB306 strain.”

6. Reviewer - “Please be more specific, literally. This is less informative than saying 'worms were fed on bacteria'”

   We edited the text (Lines 280-283) “Dried and fallen leaves from Elm and Oak trees were collected. They were autoclaved, ground by hand, and then added to the isopod containers as food, twice per week. The containers were covered with a lid with air holes. Isopods were divided into male and female chambers to prevent unwanted mating.”
